# Genome Sequences of Three Frequently Used *Listeria monocytogenes* and *Listeria ivanovii* Strains

**DOI:** 10.1128/genomeA.00404-14

**Published:** 2014-05-01

**Authors:** Jochen Klumpp, Titu Staubli, Sibylle Schmitter, Mario Hupfeld, Derrick E. Fouts, Martin J. Loessner

**Affiliations:** a Institute of Food, Nutrition and Health, ETH Zurich, Zurich, Switzerland; b J. Craig Venter Institute (JCVI), Rockville, Maryland, USA

## Abstract

We present the complete *de novo* assembled genome sequences of *Listeria monocytogenes* strains WSLC 1001 (ATCC 19112) and WSLC 1042 (ATCC 23074) and *Listeria ivanovii* WSLC 3009, three strains frequently used for the propagation and study of bacteriophages because they are presumed to be free of inducible prophages.

## GENOME ANNOUNCEMENT

*Listeria* strains are Gram-positive, rod-shaped members of the *Firmicutes* divided into nine species. *Listeria monocytogenes* is a human pathogen, whereas *Listeria ivanovii* may cause disease in animals (1). We report here the complete genome sequence of three *Listeria* strains used for studying phage-host interactions, because they are free of intact prophages (2, 3). *L. ivanovii* WSLC 3009, a serovar 5 strain, is the propagation host of various bacteriophages, such as A511. *L. monocytogenes* WSLC 1001 is a serovar 1/2a isolate (ATCC 19112) that was found to be highly similar to strain EGD (4), but it should not be confused with strain EGD-e, published in 2001. Strain WSLC 1042 is a serovar 4b isolate, frequently associated with outbreaks of infectious listeriosis (5, 6). WSLC 1001 was found not to release intact prophages, which is useful for research with and propagation of phages.

All strains were grown at 30°C in half-strength BHI medium under aerobic conditions. Genomic DNA was prepared using the Sigma genomic DNA kit. Ten micrograms of each strain’s DNA was subjected to single-molecule real-time sequencing on a Pacific Biosciences RS2 device (10-kb insert library, P4/C2 chemistry). Sequencing resulted in 54,607 reads with 5,458 kb average length for WSLC 1001, 43,186 reads with 5,059 bp average length for WSLC 1042, and 48,172 reads with 5,466 bp average length for WSLC 3009. The genomes were assembled *de novo* using SMRT Analysis 2.1.1, to 2,951,235 bp (WSLC 1001), 2,942,168 bp (WSLC 1042), and 2,919,538 bp (WSLC 3009), with 155-, 178-, and 280-fold average coverages, respectively. The genomes were annotated using the NCBI Prokaryotic Genomes Automatic Annotation Pipeline.

The WSLC 1001 genome contains 3,025 genes, 23 pseudogenes, and 67 tRNAs, the WSLC 1042 genome contains 2,945 genes, 35 pseudogenes, and 67 tRNAs, and the WSLC 3009 genome features 2,922 genes, 63 pseudogenes, and 67 tRNAs. Interestingly, using CRISPRFinder (7), WSLC 1001 and WSLC 3009 were predicted to feature two and three clustered regularly interspaced short palindromic repeat (CRISPR) loci, respectively. No CRISPRs were identified in WSLC 1042. Homologies of CRISPR spacers to different *Listeria* phages (e.g., B025, B054, P70, and A500) were found in both strains and are of particular interest regarding their putative role in the inactivation of invading bacteriophage DNA (8).

Phage_Finder (9) was used to screen the genomes for the presence of intact and cryptic phages. *L. monocytogenes* WSLC 1042 and *L. ivanovii* WSLC 3009 were found to be free of prophages. However, two phage-like regions were identified in WSLC 1001, one at positions 1 to 36557 and the other located at the end of the genome (positions 2901325 to 2944299); this second region is 42,975 bp in length and inserted into a tRNA_Arg_, a common *Listeria* prophage location (10, 11). Interestingly, both regions feature sequence homology to *Listeria innocua* phage B025 (10). Strain WSLC 1001 has one predicted monocin (12) locus at positions 1793078 to 1803804, which is 10,727 bp in length. WSLC 1042 also features a predicted monocin locus, located at positions 2483400 to 2494128, which is 10,729 bp in size. WSLC 3009 does not feature any phage or monocin-like sequence.

### Nucleotide sequence accession numbers.

The three complete genome sequences have been deposited in GenBank under accession no. CP007160, CP007210, and CP007172.
